# Fluorescently-tagged anti-ganglioside antibody selectively identifies peripheral nerve in living animals

**DOI:** 10.1038/srep15766

**Published:** 2015-10-30

**Authors:** Cynthia A. Massaad, Gang Zhang, Laila Pillai, Ali Azhdarinia, Weiqiang Liu, Kazim A. Sheikh

**Affiliations:** 1 Department of Neurology, University of Texas Health Science Center at Houston, Houston, TX 77030, USA; 2 Institute of Molecular Medicine-Center for Molecular Imaging, University of Texas Health Science Center at Houston, Houston, TX 77030, USA

## Abstract

Selective *in vivo* delivery of cargo to peripheral nervous system (PNS) has broad clinical and preclinical applications. An important applicability of this approach is systemic delivery of fluorescently conjugated ligands that selectively label PNS, which could allow visualization of peripheral nerves during any surgery. We examine the use of an anti-ganglioside monoclonal antibody (mAb) as selective neuronal delivery vector for surgical imaging of peripheral nerves. Systemic delivery of an anti-ganglioside mAb was used for selective intraneuronal/axonal delivery of fluorescent agents to visualize nerves by surgical imaging in living mice. In this study, we show that intact motor, sensory, and autonomic nerve fibers/paths are distinctly labeled following a single nanomolar systemic injection of fluorescently labeled anti-ganglioside mAb. Tissue biodistribution studies with radiolabeled mAb were used to validate neuronal uptake of fluorescently labeled mAb. Implications of this proof of concept study are that fluorescent conjugates of anti-ganglioside mAbs are valuable delivery vectors to visualize nerves during surgery to avoid nerve injury and monitor nerve degeneration and regeneration after injury. These findings support that antibodies, and their derivatives/fragments, can be used as selective neuronal delivery vector for transport of various cargos to PNS in preclinical and clinical settings.

Iatrogenic transection or injury of nerves during surgical procedures can produce substantial and sometimes chronic/irreversible patient morbidity that include sensory (numbness, chronic pain), motor (muscle weakness, cramping), or/and autonomic dysfunction (sphincteric/sexual dysfunction, ileus etc.). Nerves of small caliber and terminal nerve branches without distinct epi/perineurium that terminate in viscera and organs are particularly difficult to visualize and are more prone to damage during surgery. In this context, the development of agents for intraneuronal/axonal labeling could allow non-invasive visualization of nerves, which would be relevant for live nerve monitoring during surgical procedures to avoid nerve injury.

Current techniques for peripheral nerve labeling include intraneural or receptor/target field injections of fluorescent dyes have been used to label/visualize individual nerves^1^,^2^,^3^,^4^ but this is of limited clinical utility and more appropriate for tract tracing. Recently, *in vivo* peptide labeling of peripheral nerves was demonstrated but this technology did not distinguish between fully denervated or innervated segments of the nerves, an issue relevant to diseased nerves including nerve injuries, because of peptide specificity for connective tissue rather than neuronal/axonal binding in the nerves^5^. In this context, systemic delivery of bacterial toxins, mostly tetanus toxin or its heavy chain fragment, has been used for tract tracing^6^,^7^,^8^. Tetanus toxin heavy chain uses GT1b, a major complex ganglioside in the peripheral nervous system (PNS), for binding and internalization^8^,^9^,^10^. Although tetanus toxin labeling of the nervous system from the blood stream has been demonstrated, its further development as an *in vivo* label for live nerve imaging has not been pursued because of potential of preexisting and widespread induced immunity against these toxin fragments (heavy chain) of bacterial origin^11^.

In this technological feasibility study, we used an anti-ganglioside monoclonal antibody (mAb) with specificity for GT1b (GT1b-2b), for axonal and neuronal labeling/delivery in live animals because of vast clinical experience in the use of antibodies as therapeutic and diagnostic tools^12^,^13^,^14^. Gangliosides, the target antigens of anti-ganglioside mAb, are the predominant sialoglycoconjugates in the mammalian nervous system^15^,^16^. The most abundant gangliosides in the adult mammalian PNS, GM1, GD1a, GD1b, and GT1b are predominantly localized in neuronal/axonal compartments^16^,^17^,^18^,^19^. These glycans localize in the outer leaflet of plasma membranes where their head-groups are accessible to circulating lectins including bacterial toxins and anti-ganglioside antibodies. Gangliosides are known to constantly cycle to and from the plasma membrane by endosomal sorting, and specific bacterial toxins (cholera, tetanus, and botulinum) are known to use specific complex gangliosides and their recycling apparatus to internalize and in some cases retrogradely transport in neurons. We explored the possibility that anti-ganglioside mAb is internalized in axonal and neuronal compartments of PNS analogous to bacterial toxins. This study examined GT1b-2b mAb as selective neuronal delivery vector for intraneuronal/axonal delivery of fluorescent cargo to visualize nerves in animal studies, with implications for surgical imaging and monitoring nerve integrity and repair.

## Results

### Fluorescent tracing of peripheral nervous system

**Figure 1:**
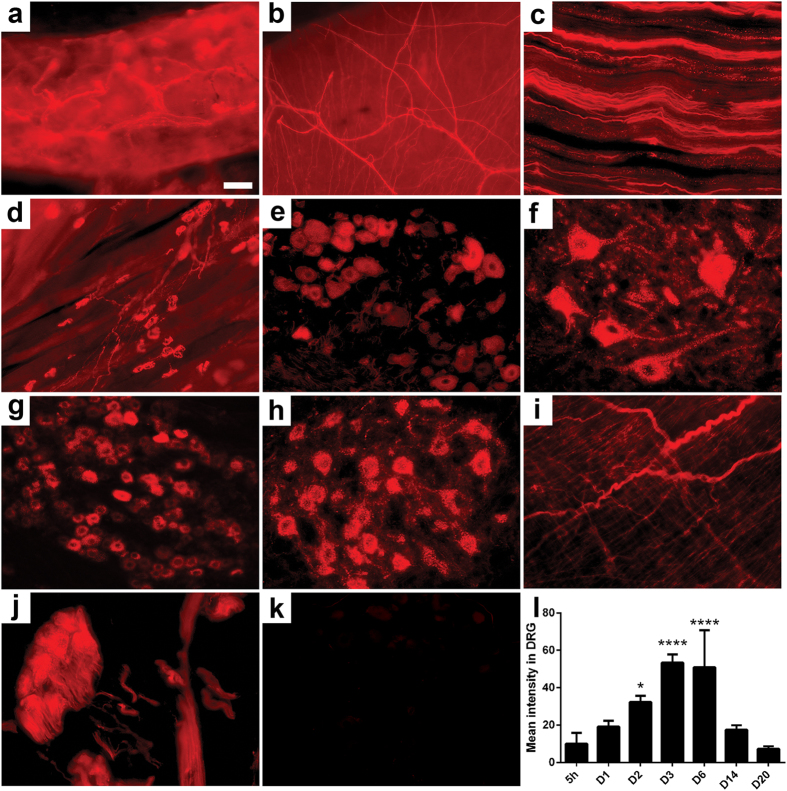
Peripheral and autonomic nervous system tracing with GT1b-2b-550. (**a**–**j**) Images taken from wild type mouse tissue at day 6 post GT1b-2b-550 injection: epidermal fibers (**a**) cornea (**b**) sciatic nerve (**c**) neuromuscular junction (**d**) DRG (**e**) ventral horn of the spinal cord (**f**) trigeminal ganglion (**g**) facial nucleus (**h**) urinary bladder (**i**) prostate gland (**j**). (**k**) DRG of mutant mice lacking complex gangliosides (*B4galnt1*-null mice) were not labeled with GT1b-2b-550. (**l**) Time course of mean labeling intensity in wild type mice DRGs. Scale bar = 50 μm. N = 4 for all groups. *p < 0.05; ****p < 0.0001.

**Figure 2:**
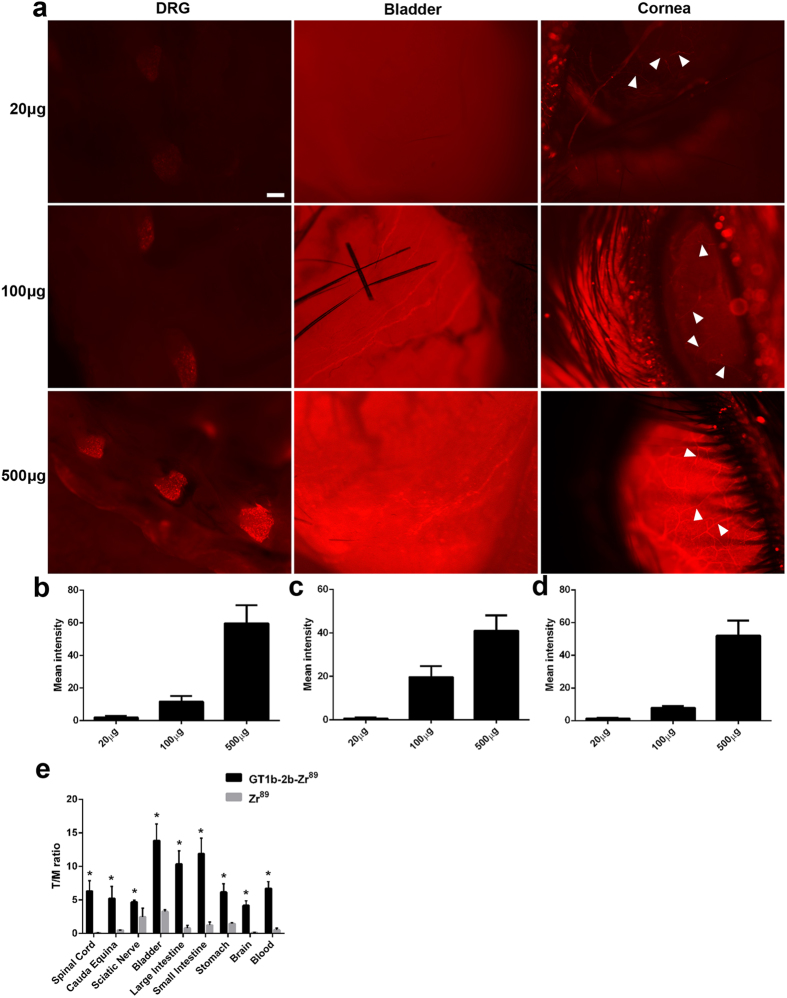
Dose response of GT1b-2b-550 binding to nerve. (**a**) Fluorescent images of DRG, bladder, and corneal nerves (arrowhead) taken from mouse receiving 20 μg, 100 μg, and 500 μg GT1b-2b-550 (corresponding respectively to 0.12, 0.6 and 3 nmoles). (**b**–**d**) Quantitative fluorescent image analysis showing DRG (**b**) bladder (**c**) and corneal nerve (**d**). Fluorescence intensity increased with higher dose of GT1b-2b-550 administered. Scale bar = 20 μm. E, Bio-distribution of GT1b2b-^89^Zr in various tissues. *p < 0.05.

Our previous studies show that GT1b-2b binds PNS neurons as well as their axons in spinal cord and DRG and peripheral nerves, respectively^19^. This mAb was labeled with fluorescent cargo (dylight550– GT1b-2b-550) and systemically administered to adult wild-type mice intraperitoneally as a single injection then tissues were harvested at various time points after antibody administration (5 hrs-21 days) for assessment of *in vivo* labeling of the PNS (including peripheral innervation of organs and viscera) and CNS by histology and microscopy (Fig. 1). Controls consisted of labeled non-specific mouse IgG injections (mIgG-550) to wild-type mice and GT1b-2b-550 injections to transgenic mice lacking complex gangliosides (Fig. 1k), both of which showed no fluorescence, indicating the specificity of GT1b-2b and ganglioside requirement for labeling. Dose response studies, tested in various organs (Fig. 2a–d), indicated that the lowest dose (20 μg) showed no staining in the DRG and bladder but stained a few fibers in the cornea. At 100 μg, the staining is clearly visible in all organs, however at 500 μg, the staining was the clearest. Based on these findings 500 μg (or 3 nmoles) was used for all subsequent studies.

Our studies clearly showed specific neuronal distribution of fluorescent antibody in various tissues upon systemic injection. To confirm the specificity of our antibody and obtain quantitative analysis of *in vivo* biodistribution, GT1b2b was radiolabeled with ^89^Zr and administered systemically to mice. The long half-life of ^89^Zr permitted delayed analysis of GT1b2b biodistribution, and various tissues were collected 4 days after injection and assessed for radioisotope content. Radiotracer uptake in tissues of interest were normalized to muscle uptake and compared to a non-targeted ^89^Zr control. In line with live fluorescent imaging where the signal from viscera was very high, the bladder, large and small intestines and stomach contain the highest levels of radioactivity. A significant increase in radioactivity is also noted in the brain, spinal cord and spinal nerves in the cauda equina, confirming the results from the live fluorescent imaging (Fig. 2e).

**Figure 3:**
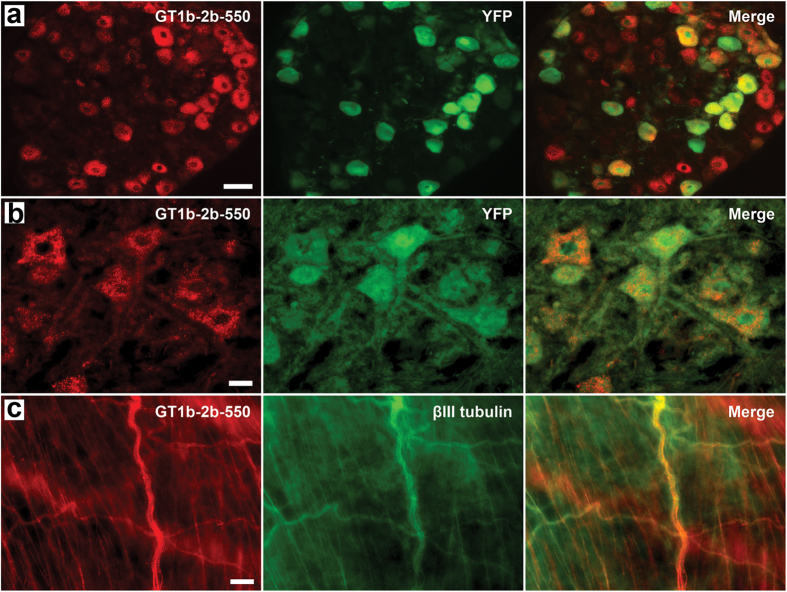
The validation of neuronal/axonal labeling by GT1b-2b-550. (**a**,**b**) Co-localization of GT1b-2b-550 and YFP signal in DRG (**a**) and anterior horn spinal neurons (**b**) in Thy1-YFP mice. (**c**) GT1b-2b-550 labeled the visceral nerves (βIII tubulin positive) in wild-type mouse bladder wall. Scale bar = 50 μm (**a**) or 20 μm (**b**,**c**).

**Figure 4:**
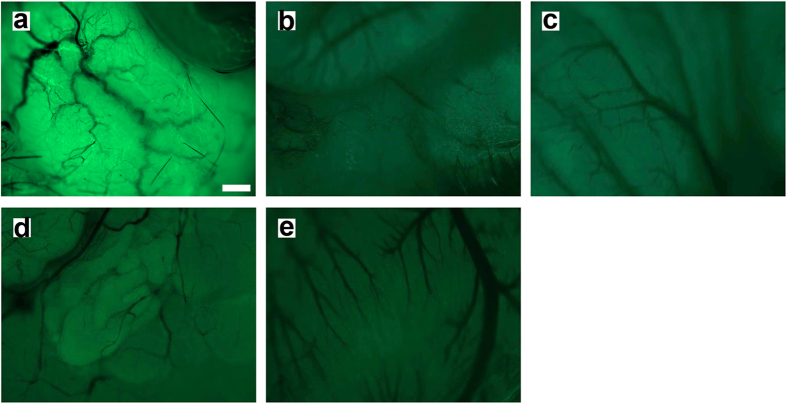
Thy1-YFP live imaging in the viscera. Images taken from a live anesthetized mouse: (**a**) Urinary bladder; (**b**) Stomach; (**c**) Intestine; (**d**) Prostate gland; (**e**) Heart. Scale Bar = 20 μm.

**Figure 5:**
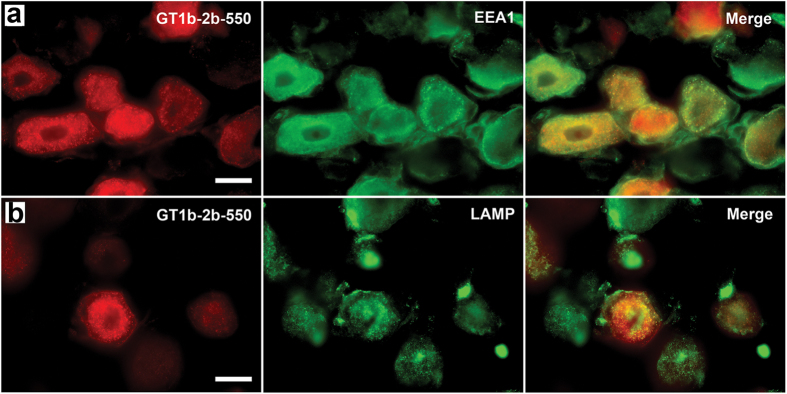
GT1b-2b-550 was up-taken by neuronal cells and found in early and late endosomes. (**a**,**b**) Labeled GT1b-2b (GT1b-2b-550) can be found in the neuronal cellular compartments, which are identified as early (**a**) and late (**b**) endosomes by anti-EEA1 and anti-LAMP antibodies, respectively. Scale bar = 20 μm.

The neuronal and axonal labeling by GT1b-2b-550 was further validated in the transgenic Thy1-YFP mice (Fig. 3), in which yellow fluorescence protein (variant of GFP) is exclusively expressed in neurons and axons^20^. The co-localization between GT1b-2b signal and YFP was high for sensory and motor neurons and their axons. GT1b-2b had more labeling of different neuronal populations compared to YFP, for example, virtually all DRG neurons were labeled by GT1b-2b but a number of these neurons did not express YFP, as reported previously^20^. Notably, most of the nerve plexuses around abdominal viscera did not express YFP (Fig. 4), whereas GT1b-2b strongly labeled these visceral nerves. The labeling of visceral nerves by GT1b-2b-550 was confirmed in wild-type mice (see below). Immunocytochemistry with endosomal markers showed that the GT1b-2b co-localizes with early and late endosomes (Fig. 5). This is consistent with a previous *in vitro* study^21^ showing that internalized anti-ganglioside antibodies co-localize with early and late endosomal markers in PC12 cells and cultured primary DRG neurons.

### Fluorescent labeling and surgical imaging of peripheral nerves

**Figure 6:**
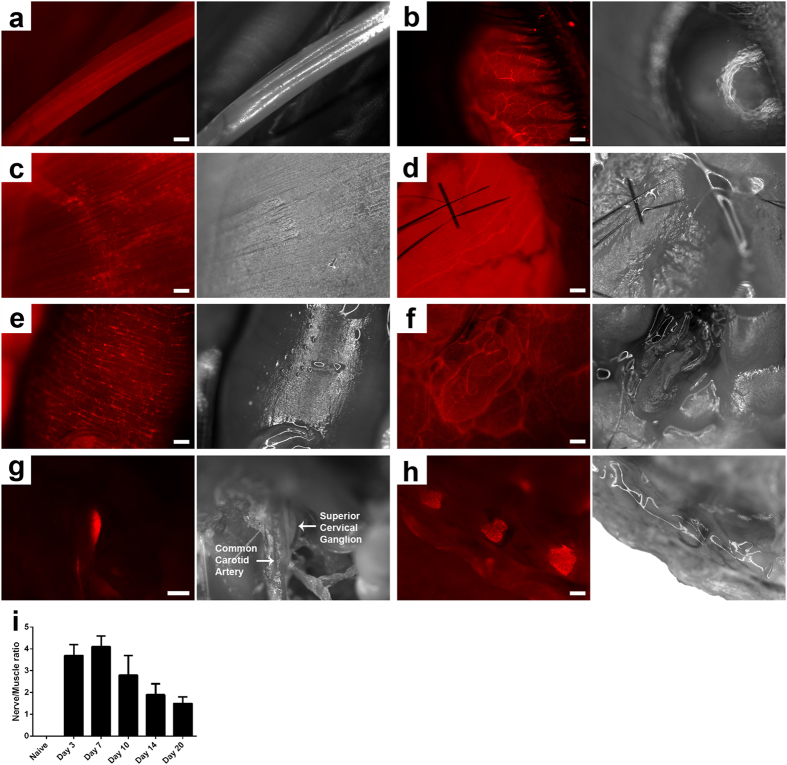
Live labeling and monitoring of peripheral nerves in mice with GT1b-2b-550. (**a**–**h**) Fluorescent and corresponding brightfield images taken from whole mouse at day 6 post GT1b-2b-550 injection. (**a**) Sciatic nerve; (**b**) Cornea; (**c**) Neuromuscular junction; (**d**) Urinary bladder; (**e**) Intestines; (**f**) Prostate; (**g**) Superior cervical ganglion; (**h**) DRG. (**i**) Contrast ratios of nerve to muscle calculated from sciatic nerve at multiple time points post GT1b-2b-550 injection. Scale bar = 10 μm (**c**) or 20 μm (A; B; D; E; F; H), or 50 μm (**g**).

Living and post-fixed *ex vivo* imaging was performed at time points ranging from 24 hours to 21 days after GT1b-2b-550 injection. In selected studies corneal labeling was examined from 5hrs to 21 days after GT1b-2b-550 injection. Signal in peripheral nerves started to appear 24 hrs after antibody injection and peaked on day 6 post-injection. The contrast between nerves and surrounding tissue (mostly muscles) was very clear with fluorescent macroscopy (7–25X magnifications). GT1b-2b-550 labeling allowed visualization of all major peripheral nerves, their small branches, and motor innervation of thin strap muscles such as intercostals. Labeling was more pronounced in cell bodies and nerve termini than in peripheral nerve trunks. Mixed (sciatic and tibial), and predominantly motor (phrenic) nerves were equivalently labeled. Contrast ratios between the nerve and muscle were calculated for the sciatic nerve (Fig. 6). Useful contrast developed 24 hrs after injection, peaked on days 3–6 post-injection, started to decline on post-injection day 10 and later time points. Live macroscopic studies also visualized the entire extent of sciatic nerve and its terminal branches. These findings likely reflect simultaneous exposure, uptake, and retrograde transport of systemically administered antibody by short and long terminal branches. This is consistent with tracing/histological studies showing labeling of the entire extent of sciatic nerve, its terminal branches, and parent neurons in the spinal cord and DRGs 24 hrs post-injection. Our current studies did not show any GT1b-2b-mediated nerve fiber damage in sciatic nerves as assessed by electrophysiology and morphological and morphometric analysis of epon embedded nerves (Supplementary Fig. 1).

Whole nerve/animal imaging findings were consistent with histological findings. Overall, still images were easier to acquire *ex vivo* compared to macroscopy on live animals due to breathing motion. However, dynamic/video capturing was less affected by breathing motion and shows the feasibility of this application to larger animals and/or clinical settings (Supplementary videos 1,2,3). Additionally, acquisition of brightfield images required using a direct light shining on the live mouse. Due to the 3D nature of the specimen, some light refraction/reflection occurred, as seen on all brightfield images included in the manuscript. Fluorescent images/video acquisition however was not affected.

Similar to tracing studies (as above), live imaging showed widespread labeling of autonomic nerve fibers in the abdominal and pelvic viscera. Live imaging was performed on these viscera on days 6 and 14 after a single i.p. injection of GT1b-2b-550 because nerve fiber staining peaked at day 6 and disappeared on day 14 after the antibody injection. We found strong labeling of the hollow viscera like the stomach and small and large intestines (Fig. 6, Supplementary videos 1 and 2), and the urinary bladder (Fig. 6 and Supplementary video 3). Labeling of the small visceral bundles surpassed that of larger somatic peripheral nerves. Neuronal networks innervating the small and large intestines were clearly delineated by macroscopy (7–20X). Beneath the intestines, para-aortic autonomic nerve fiber bundles/plexuses were also visible. The urinary bladder had the most intense labeling/signal, amongst abdominal and pelvic viscera, which precluded recognition of individual nerve bundles at the 500 μg dose. At the lower dose of 100 μg, staining was less intense and therefore single fibers were visible. The prostate gland was also imaged by retracting the bladder and the neural network on its surface was visualized by live macroscopy (Fig. 6). Live imaging of the thoracic compartment was limited due to the rapid demise of animals with opening of the thoracic cavity but we were able to image nerve fiber bundles on the surface of the heart.

### Monitoring peripheral nerve injury and repair by selective neuronal delivery vector-based fluorescent surgical imaging

**Figure 7:**
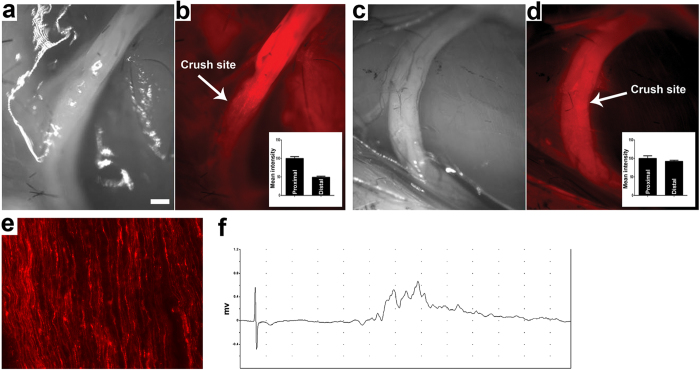
Live labeling and monitoring of nerve injury and regeneration in mice with GT1b-2b-550. (**a**,**b**) Sciatic nerve 4 days following nerve crush and 7 days following GT1b-2b-550 administration. Brightfield image taken live from the surgical field (**a**) corresponding fluorescent image distinctly showing the crush site as discontinuous labeling between the proximal and distal stumps of the nerve (**b**). Inset shows quantification of labeling in the proximal versus the distal portion of the nerve. (**c**,**d**) Sciatic nerve 21 days following nerve crush and 4 days following GT1b-2b-550 administration. Brightfield image taken live from the crush surgical field (**c**) corresponding fluorescent image showing labeling of the entire nerve, which indicates labeling of the regenerating fibers in the distal portion of the nerve (**d**). Inset shows quantification of labeling in the proximal versus the distal portion of the nerve. (**e**) Micrograph showing the individual regenerating nerve fibers labeled by GT1b-2b-550 on day 21 post sciatic nerve crush. (**f**) Delayed and dispersed CMAP amplitude recorded on day 21 post sciatic nerve crush is consistent with target/muscle re-innervation after nerve injury. Scale bar = 20 μm.

Nerve crush in experimental animals is representative of the traumatic nerve injury in continuity and allows the assessment of axon degeneration and regeneration in the distal stump. Since GT1b-2b distinctly labels axons in peripheral nerves, we examined whether systemic delivery of GT1b-2b-550 can detect axon degeneration and regeneration in the distal stumps of injured nerves in continuity. To study axon degeneration in the distal stumps of injured nerves mice were injected with GT1b-2b-550 on day 0 and on day 3 post-injection sciatic nerves were imaged in live animals to confirm nerve labeling. At this time sciatic nerves were also crushed and live animals were reimaged 4 days after nerve injury (day 7 after GT1b-2b-550 administration). We found that nerve fibers were still labeled in the proximal stump, whereas the labeling was lost in the distal stump in which all nerve fibers degenerate (Fig. 7b).

To examine axon regeneration in distal stumps of nerves with injury in continuity, sciatic nerves were crushed on day 0. GT1b-2b-550 was injected on day 17 and live animal imaging was performed on day 21 after nerve crush. In this model, on day 17 post nerve crush, significant axon regeneration occurs in the distal sciatic stump and its terminal branches including tibial nerve, and there is reinnervation of hind paw muscles, as confirmed by electrophysiology and morphological studies^22^. We found that newly regenerated fibers in the distal stump of sciatic nerve and its branches were labeled with GT1b-2b-550 (Fig. 7d,e). The live imaging findings, indicating labeling of regenerated axons in the distal portion/branches of the sciatic nerve, were also confirmed by electrophysiology (Fig. 7f).

## Discussion

This proof of concept study demonstrates that ligands for complex gangliosides are useful for labeling peripheral nerves and nerve plexuses around viscera that can be monitored by fluorescent macroscopy in live animals during surgical exposure. We also develop an application of fluorescent live labeling with anti-ganglioside mAb, which allows identification of nerve segments with axonal degeneration and regeneration by nerve macroscopy in living animals. Currently, nerve integrity during surgery is monitored by intraoperative electrophysiology but this technique has limitations, as it only monitors nerve integrity without providing visual guidance to the surgeon to avoid accidental injury to the nerve^23^,^24^,^25^,^26^,^27^. Accordingly, development of live imaging tools, such described in this paper, are needed. Further, this live labeling technique can be used to visualize nerves in virtually all strains of mice expressing normal repertoire of complex gangliosides and this labeling is not limited to transgenic mice expressing variants of flourescent proteins^20^.

Our macroscopy data, in conjunction with tracing/histological studies, indicate that the labeling of a proportion of axons in the nerve trunks is sufficient to provide useful contrast ratios to visualize nerve bundles during surgical exposure. Systemic delivery and associated simultaneous labeling of short and long axons/terminal nerve branches and the need for only a proportion of nerve fiber labeling to macroscopically visualize nerve trunks are features that overcome limitations of locally administered neuronal tracers. The neuronal tracers only label selected nerve fibers of relatively uniform length innervating injected targets and labeling is also dependent on the rate of axonal transport. Based on the labeling characteristics of anti-ganglioside mAb we anticipate that systemic delivery of these reagents would allow macroscopic visualization of nerve trunks/bundles in longer nerves in larger animals with comparable labeling and visualization kinetics as in mice.

This study shows the feasibility of using anti-GT1b-2b mAb to assess peripheral nerve degeneration and regeneration in sciatic nerve crush model (traumatic nerve injury in continuity). This is relevant clinically as there is an immediate need for reliable measures to assess peripheral nerve integrity and regeneration in patients with traumatic nerve injuries in continuity. Current clinical and investigative tools used to assess these disorders are severely limited by their complete dependence on target connectivity and virtual inability to provide information once disconnected {reviewed in^28^}.

We show that labeled GT1b-2b mAb has superior labeling of cell bodies and nerve termini compared to peripheral nerve trunks. Small visceral bundles are also better labeled than large somatic peripheral nerves. This variation in labeling of polarized neuronal cells could be due to multiple factors such as differences in: volume of distribution between large and small nerves; blood/nerve barrier between somatic and visceral nerves; distribution and accumulation of antibody-containing vesicles in axonal compartments versus neuronal soma; and differences in ganglioside/antigen distribution between somatic and visceral nerves. Moreover, nerve termini are more accessible to the labeled circulating anti-ganglioside mAb and likely serve as portals of antibody entry.

In current studies, we found that the labeled anti-ganglioside mAb can be taken up by neurons and enter endosomal and lysosomal compartments, which prevents the antibody from causing nerve injury at the cell surface as proposed previously^21^. Anti-ganglioside antibodies can induce nerve fiber injury and previous work indicates that this antibody-mediated nerve injury is entirely dependent on interactions of Fc portion of these antibodies with effectors of innate immune system including complement and Fc gamma receptors (FcγRs)^29^,^30^,^31^. We found that inflammatory milieu in the nerve is prerequisite for anti-ganglioside antibody-induced nerve injury models and activating FcγRs-mediated inflammation plays a pivotal role in producing neuropathic effects^29^,^31^. Notably, anti-ganglioside antibodies had no pathological effects on intact/uninjured nerves in our models^29^,^31^, which is again reconfirmed in this study. Further, modification of the Fc portion with antibody-engineering^32^,^33^,^34^ can allow development of reagents that do not interact with effectors of innate immunity, thus precluding this potential risk.

Our data support the notion that anti-ganglioside antibodies can be used as vectors for selective *in vivo* delivery of cargo to PNS neuronal and axonal compartments. Recent advances in biotechnology have allowed generation of specific bioactive molecules including enzymes, proteins, peptides, and small molecules that can enhance neuronal regeneration in cell culture studies. However, targeted delivery/transportation of these agents due to the need to cross blood-tissue and/or extracellular-cellular barriers is extremely inefficient. Selective neuronal delivery vectors have the potential to target bioactive (neuroprotecitve and/or proregenerative) molecules to appropriate cellular (particularly post-mitotic neurons) and subcellular compartments. Such targeted ligand delivery can be much more efficient in improving bioavailability and limiting off-target effects (nonneuronal tissues/cells) that can not only cause systemic side effects but more importantly hamper the repair process.

In summary, this biotechnological study shows the feasibility of using an anti-ganglioside mAb for intraneuronal labeling of peripheral nerves for viewing with fluorescent cameras during: a) various surgical procedures to avoid injuries to small nerve bundles; and b) surgical repair of injured peripheral nerves.

## Materials and Methods

### Mice

Adult male C57BL/6, Thy1-YFP mice (Jackson labs, Bar Harbor, ME) and *B4galnt1*-null (GM2/GD2 synthase/*GalNAcT*-null; in house colony) mice were used^35^. Wild-type C57BL/6 mice express a normal repertoire of gangliosides in their nervous system. *B4galnt1*-null animals express only simple gangliosides GM3 and GD3 but not complex gangliosides like GM1, GD1a, GD1b or GT1b in their nervous system^19^,^36^. These animals were used to confirm that the uptake and internalization of anti-ganglioside antibodies is ganglioside dependent. All studies were performed according to institutional guidelines and animals were handled according to protocols that were approved by the Animal Welfare Committee at the University of Texas Health Science Center at Houston (Protocol number: HSC-AWC-14-067) and that are in accordance with Federal guidelines. All animals were fed ad libitum and housed according to standard university policy. For various studies described below 3–5 animals/study group were used.

### Anti-ganglioside mAb fluorophore labeling

Anti-ganglioside antibody generation, production, and purification was previously described^37^. GT1b-2b mAb were labeled with amine-reactive Dylight550 following manufacturer’s instructions (Thermofisher Pierce, Rockford IL). In select studies, a commercially available mouse IgG (Equitech Bio Inc., Kerrville TX) was similarly labeled with Dylight 550 and used as a control (CmIgG-550).

### Histology and immunocytochemistry

Animals were transcardially perfused with cold PBS and lightly fixed in 4% paraformaldehyde. Multiple organs were dissected, cryopreserved in 30% sucrose/PBS and then cryosectioned at 10 μm. Some sections were immediately mounted using Vectashield containing DAPI (Vector labs, Burlingame, CA) and the other parts were processed for further immunohistochemistry. Briefly, sections were blocked in PBS containing 1% BSA, 10% normal goat serum and 0.3% triton X-100. Then they were incubated with primary antibodies as indicated, diluted in PBS containing 1% BSA, 1% normal goat serum and 0.1% triton X-100. Following washing with PBS, sections were incubated for 1 hour with an appropriate secondary antibody conjugated to a green fluorophore (to distinguish from the red dylight550). Secondary antibodies were diluted in PBS containing 1%BSA and 1% normal goat serum. Primary antibodies and dilutions: 1:1000 anti-EEA1 (Early Endosome Antigen 1; Early Endosome Marker; Santa Cruz Biotechnology, Dallas, TX) and 1:1000 anti-LAMP1 (Lysosomal-associated Membrane Protein 1; Late Endosome Marker; Abcam, Cambridge, MA), 1:1000 anti-BIII tubulin (Promega, Madison, WI), and 1:200 anti-neurofilament (Sigma-Aldrich Corp., St. Louis, MO). Secondary antibodies and dilutions: 1:500 Goat anti-mouse IgG-FITC and 1:500 Goat anti-rabbit IgG-FITC (Jackson immunoresearch, West Grove, PA). The latter immunostaining was performed to both confirm nerve labeling and identify location in subcellular compartments.

### *In vivo* fluorescent labeling and monitoring

Fluorescently labeled anti-ganglioside antibodies were injected intraperitoneally at doses ranging from 0.1 to 3 nmoles. Controls were injected with a similar dose of mIgG-550. *In vivo* monitoring started 5 hours post-injection and lasted up to 21 days post-injection. At selected time points, mice were anesthetized with 2% isoflurane in oxygen and imaged live with a Zeiss Axiozoom V16 fluorescent macroscope (Carl Zeiss microscopy, LLC). The macroscope base was modified with a surgical heated table that allows anesthetizing animals and keeping them warm for live imaging. Standard excitation/emission spectra were utilized for the different fluorophores: 562/576 nm for dylight 550 and 488/518 nm for green fluorescent protein (GFP). Quantitative fluorescent image analysis was performed by measuring the fluorescent signal intensity of region of interest with background noise subtraction.

### Radio-labeling studies

For radioactive studies, GT1b-2b mAb was labeled with the positron-emitting radioisotope, Zirconium-89 (^89^Zr, t_1/2_ = 3.3 days), using published methods (Ikotun O.F., *et al.*, PLoS One 2013, PMID 24143237) with modifications. Briefly, GT1b-2b mAb (1 mg) was reacted with a 6-fold molar excess of deferoxamine-p-SCN (DFO, Macrocyclics, Dallas, TX) in 0.1 M sodium carbonate overnight at 4 °C. The reaction was purified with Zeba desalting spin columns (Thermo Scientific, Rockford, IL) and immunoreactivity was confirmed by ganglioside ELISA. Sample purity was assessed by size-exclusion HPLC and demonstrated no changes in the integrity of the immunoconjugate. ^89^Zr-oxalate (Washington University-St. Louis, MO) was diluted with 0.5 M HEPES (4-(2-hydroxyethyl)-1-piperazineethanesulfonic acid) and neutralized to pH 7.4 using 2 M sodium hydroxide. For radiolabeling, 1 mCi of ^89^Zr was added to a solution containing 100 μg of DFO-GT1b2b in 1 M HEPES, and the reaction was incubated for 1 hour at 37 °C. To chelate any unreacted radioisotope, EDTA (ethylenediaminetetraacetic acid) was added to the reaction mixture and incubated for an additional 15 minutes. Following Zeba purification, ^89^Zr-DFO-GT1b2b was collected in PBS with radiochemical yields of 90.2 ± 3.2% (n = 4) and radiochemical purity >95% as determined by radio-thin-layer chromatography and radio-HPLC.

For the animal studies, 40 μg (0.27 nmol) of ^89^Zr-DFO-GT1b2b was administered intravenously (i.v.) to each mouse. Control animals received free ^89^Zr. Animals were euthanized at 90–96 hours post-injection and selected tissues were resected, weighed, and counted using a Wizard2 gamma counter (Perkin Elmer, Waltham, MA) to quantify tissue uptake. The results were expressed as percentage of the injected dose per gram of tissue (%ID/g) as well as tissue-to-muscle (T/M) ratios generated by normalizing various tissue radioactivity uptakes to muscle signal.

### Sciatic nerve injury

All sciatic nerve crush studies were done on 8 to 10-week-old wild type C57BL6 mice. A standardized sciatic nerve crush model was used, as previously described^22^. Briefly, right sciatic nerves were crushed 35 mm rostral to the middle toe using fine forceps. Separation of the proximal and distal endoneurial contents indicated complete crush. To study axon degeneration in the distal stumps of injured nerves mice were injected with GT1b-2b-550 on day 0 and on day 3 post-injection sciatic nerves were imaged in live animals to confirm nerve labeling. At this time sciatic nerves were also crushed and live animals were reimaged 4 days after nerve injury (day 7 after GT1b-2b-550 administration). To examine axon regeneration in the distal stumps of nerves with injury in continuity, sciatic nerves were crushed on day 0. GT1b-2b-550 was injected on day 17 and live animal imaging was performed on day 21 after nerve crush. In this model, on day 17 post nerve crush, significant axon regeneration occurs in the distal sciatic stump and its terminal branches including tibial nerve, and there is reinnervation of hind paw muscles, as confirmed by electrophysiology and morphological studies^22^.

### Toxicity and motor function

To ensure that dose and formulation of anti-ganglioside antibody used (GT1b-2b-550 or GT1b-2b-Gd) is not toxic to intact nerves, electrical and morphological studies were carried out in adult male C57BL/6 injected with a single 3 nmole dose intraperitoneally, similar to the highest dose used for the imaging studies. Electrical studies included measurement of compound muscle action potential amplitude and nerve conduction latency, as described previously^22^. Morphological studies included histological assessment of nerve integrity. Sciatic nerve and tibial nerve segments were embedded in Epon, then 1 μm toluidine blue stained sections were obtained and one entire section of the whole nerve segment/animal was used for quantification, as described previously^35^,^38^. All myelinated axons in a single whole cross section of the nerve were counted at light level (40X) by using a motorized stage and stereotactic imaging software (Axiovision; Zeiss), as described^22^. This morphometric method avoids random sampling and includes all fibers in analysis.

### Statistics

ANOVA with corrections for multiple comparisons was used to analyze the data with normal distribution (Normality was determined by D’Agostino-Pearson omnibus test); otherwise, Kruskal-Wallis nonparametric test was performed. *p* values < 0.05 were considered statistically significant. Sample sizes are based on our previous studies with anti-ganglioside mAbs^22^ and similar to those generally employed in the field.

## Additional Information

**How to cite this article**: Massaad, C. A. *et al.* Fluorescently-tagged anti-ganglioside antibody selectively identifies peripheral nerve in living animals. *Sci. Rep.* **5**, 15766; doi: 10.1038/srep15766 (2015).

## Supplementary Material

Supplementary Information

Supplementary Video 1

Supplementary Video 2

Supplementary Video 3
