# Supplementary material for: Fluorescently-tagged anti-ganglioside antibody selectively identifies peripheral nerve in living animals
Source: Sci Rep. 2015 Oct 30;5:15766. doi: 10.1038/srep15766 (PMC4626805; doi:10.1038/srep15766)

Fluorescently-tagged anti-ganglioside antibody selectively identifies peripheral nerve in living animals

Cynthia A. Massaad[1], Gang Zhang[1], Laila Pillai[1], Ali Azhdarinia[2], Weiqiang Liu[1], and Kazim A. Sheikh[1*]

[1]Department of Neurology, [2]Institute of Molecular Medicine-Center for Molecular Imaging, University of Texas Health Science Center at Houston, Houston, TX 77030, USA

Supplementary figure and video legends:

Supplementary figure 1: GT1b-2b-550 did not induce nerve injury. Micrographs of sciatic nerves (a-c); sciatic nerve of a wild type mouse without antibody injection (a); sciatic nerve of a wild type mouse injected with control mouse IgG (CmIgG-550) (b); sciatic nerve of a wild type mouse injected with GT1b-2b-550 (c). Morphometric (d) and electrophysiological (e) analyses indicate that GT1b-2b-550 did not induce nerve injury in wild type mice. Scale bar = 10μm.

Supplementary video 1: live imaging in the stomach. Video acquired from a live anesthetized mouse on day 6 post GT1b-2b-550 injection. Shown here is the stomach at 20X magnification. Note the clearly labeled nerve network.

Supplementary video 2: live imaging in the small intestine. Video acquired from a live anesthetized mouse on day 6 post GT1b-2b-550 injection. Shown here is the small intestine at 20X magnification. Note the clearly labeled nerve network.

Supplementary video 3: live imaging in the bladder. Video acquired from a live anesthetized mouse on day 6 post GT1b-2b-550 injection. Shown here is the bladder at 7X magnification. Although individual nerves are not visible at this magnification, bladder labeling is very intense and clearly contrasted from neighboring structures.

Supplementary Figure 1

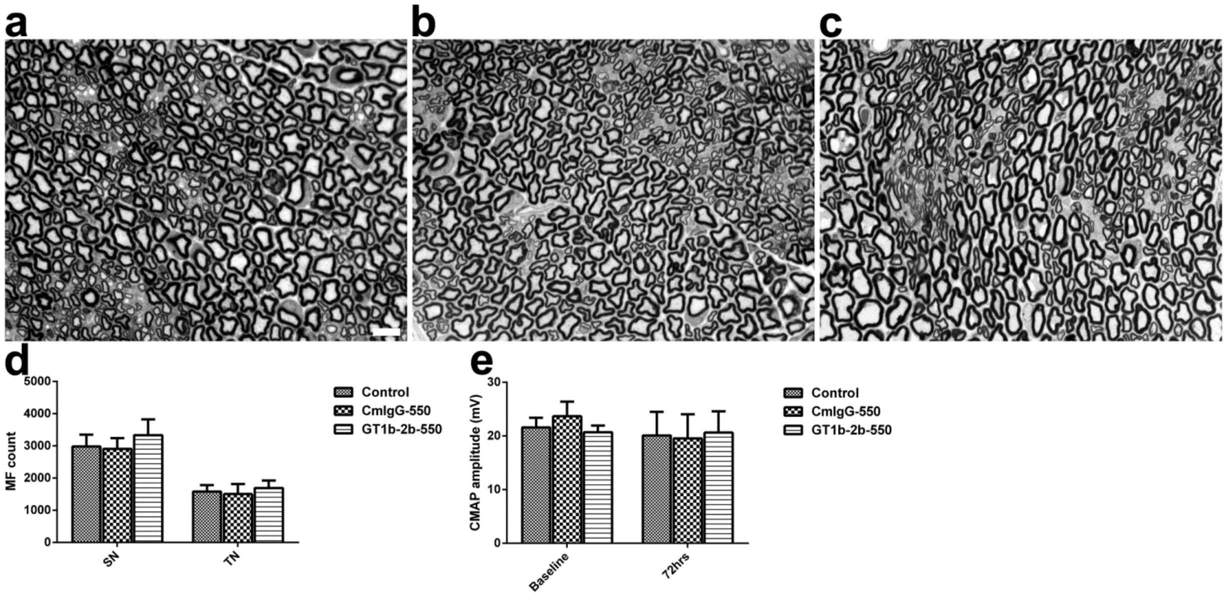

Supplement: Supplementary Information [file srep15766-s1.pdf]
